# Predictors of Mild Cognitive Impairment Stability, Progression, or Reversion in the Lothian Birth Cohort 1936

**DOI:** 10.3233/JAD-201282

**Published:** 2021-03-09

**Authors:** Miles Welstead, Michelle Luciano, Graciela Muniz-Terrera, Stina Saunders, Donncha S. Mullin, Tom C. Russ

**Affiliations:** a Lothian Birth Cohorts, School of Philosophy, Psychology & Language Sciences, University of Edinburgh, Edinburgh, UK; b Edinburgh Dementia Prevention, University of Edinburgh, Edinburgh, UK; c Alzheimer Scotland Dementia Research Centre, University of Edinburgh, Edinburgh, UK; d Division of Psychiatry, Centre for Clinical Brain Sciences, University of Edinburgh, Edinburgh, UK

**Keywords:** Aged, cognitive dysfunction, memory, public health

## Abstract

**Background:**

Mild cognitive impairment (MCI) describes a borderland between healthy cognition and dementia. Progression to and reversion from MCI is relatively common but more research is required to understand the factors affecting this fluidity and improve clinical care interventions.

**Objective:**

We explore these transitions in MCI status and their predictive factors over a six-year period in a highly-phenotyped longitudinal study, the Lothian Birth Cohort 1936.

**Methods:**

MCI status was derived in the LBC1936 at ages 76 (*n* = 567) and 82 years (*n* = 341) using NIA-AA diagnostic guidelines. Progressions and reversions between healthy cognition and MCI over the follow-up period were assessed. Multinomial logistic regression assessed the effect of various predictors on the likelihood of progressing, reverting, or maintaining cognitive status.

**Results:**

Of the 292 participants who completed both time points, 41 (14%) participants had MCI at T1 and 56 (19%) at T2. Over the follow-up period, 74%remained cognitively healthy, 12%transitioned to MCI, 7%reverted to healthy cognition, and 7%maintained their baseline MCI status. Findings indicated that membership of these transition groups was affected by age, cardiovascular disease, and number of depressive symptoms.

**Conclusion:**

Findings that higher baseline depressive symptoms increase the likelihood of reverting from MCI to healthy cognition indicate that there may be an important role for the treatment of depression for those with MCI. However, further research is required to identify prevention strategies for those at high risk of MCI and inform effective interventions that increase the likelihood of reversion to, and maintenance of healthy cognition.

## INTRODUCTION

Mild cognitive impairment (MCI) is used to describe individuals presenting with cognitive decline above what would be expected of normal ageing but not severe enough to warrant a diagnosis of dementia [1, 2]. Prevalence of MCI in older adults for individuals in their 70s and 80s is typically reported to be 10–25%[3–5]. However, these MCI prevalence rates are cross-sectional and do not account for changes over time. Overton et al. [6] report that MCI status is not stable and both progression to and reversion from an MCI state can occur. Thus, longitudinal studies with repeated measures have particular value.

Progression from healthy cognition to MCI is common and well documented in the longitudinal literature [7, 8]. It has been established that the cognition of those with MCI frequently remains stable [9]. Reversion from MCI to healthy cognition is also relatively frequent, with several studies reporting that those with MCI are more likely to revert to healthy cognition than progress to dementia [6, 10, 11]. Subsequent research has focused on understanding why certain individuals progress to, revert from, or maintain MCI whereas others do not. Higher rates of MCI conversion and maintenance are associated with a plethora of factors including age, education, race, cardiovascular risk factors, diabetes, depression, *APOE* *ɛ*4 status, Parkinson’s disease, and sleep disorders [6, 12–17].

Previous research into transition from normal cognition to MCI is inconsistent and focused primarily on either progression or reversion rather than considering them both simultaneously. Accordingly, further research, which considers transitions both to and from an MCI state, may allow for better data synthesis in the future. By understanding the key factors associated with MCI state transitions, it may be possible to improve the ability for interventions to lessen an individual’s risk of developing MCI, but also to facilitate reversion from it or stability in those who already have it. Here we explore the predictive factors which are associated with MCI stability, progression to MCI, and reversion from MCI over approximately six years in the Lothian Birth Cohort 1936 (LBC1936) [18, 19]. One of the key differences between the LBC1936 and other longitudinal cohorts used in this field is that all participants were born in 1936 in Scotland. Accordingly, due to the narrow age gap and similar geographical area, participants have had similar life experiences, for instance, living through the introduction of a National Health Service or World War II. Thus, the homogeneity of their experiences makes them more suitable for modelling some aspects of ageing compared to a broader sample whereby cohort effects may complicate such analyses. Additionally, the wealth of information collected by the study enables us to explore MCI transitions while controlling for not only a range of previously researched biopsychosocial factors, but also novel risk factors such as age 11 cognitive function. Using this wealth of information, we hypothesize that several risk factors will emerge that influence MCI stability over follow-up.

## METHODS

### Study sample

The LBC1936 study consists of 1091 participants, almost all born in 1936 with a mean age of 69.5 years (SD = 0.9) at recruitment. Wave 1 took place from 2004–2007, with follow-up visits approximately every three years thereafter (wave 2 *n* = 866, wave 3 *n* = 697, wave 4 *n* = 550, wave 5 *n* = 431). For more details on recruitment and testing procedures, see [19, 20]. MCI status could only be determined at Wave 3 (mean age [SD] = 76.3 [0.7]) and Wave 5 (mean age [SD] = 82.1 [0.5]) allowing a follow-up period of approximately six years. The LBC1936 study was conducted according to the Declaration of Helsinki guidelines. Ethical permission for the LBC1936 study protocol was obtained from the Multi-Centre Research Ethics Committee for Scotland (Wave 1: MREC/01/0/56), the Lothian Research Ethics Committee (Wave 1: LREC/2003/2/29), and the Scotland A Research Ethics Committee (Waves 2, 3, 4, & 5:07/MRE00/58). Written consent was obtained from participants at each of the waves.

### MCI coding

**Fig. 1:**
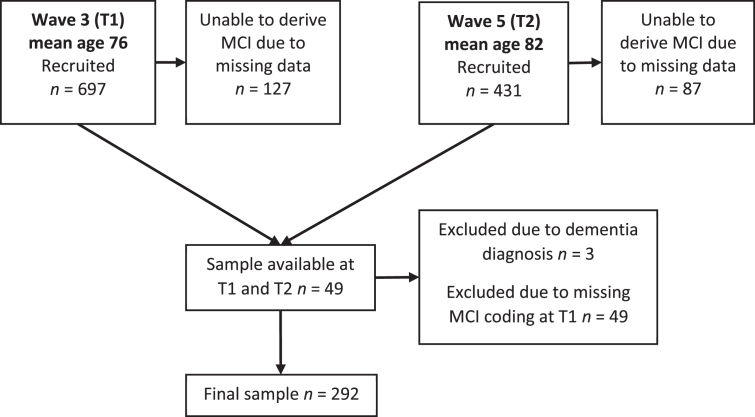
A flow chart to demonstrate how many participants were recruited and assessed at each time point.

MCI was coded in the LBC1936 according to the criteria outlined by the National Institute on Aging-Alzheimer’s Association (NIA-AA) workgroups on diagnostic guidelines for Alzheimer’s disease [1]. Accordingly, MCI was based on subjective concern regarding a change in cognition, impairment in one or more cognitive domains, preservation of independence in functional abilities, and no diagnosis of dementia. Further detail has been previously reported [21]. In the following analyses, MCI codings were used at Wave 3 (T1) & Wave 5 (T2). Missing data meant that not all participants received an MCI coding. Additionally, of those participants who had MCI coding at T2, 49 had missing data at T1 and accordingly were excluded as this prevented the calculation of change in MCI status over time. Accordingly, MCI was coded for 567 participants at T1 and 292 at T2. Figure 1 illustrates the number of participants recruited, assessed, followed-up and excluded, at both time points.

### Covariates

T1 predictors of MCI change over the follow-up period were chosen based on previous research showing associations between them and cognitive decline [16, 22–25]. These included: age at T1 (range = 74.59–77.70), sex, years of full-time education, age 11 cognitive function (calculated from the Moray House Test score at age 11 years [26] and standardized for age in days on the test-date), occupational social class (professional/mana-gerial/skilled, non-manual/skilled manual or semi-skilled/unskilled), *APOE* *ɛ*4 status (gene present/absent), self-reported history of cardiovascular disease (coded yes or no), number of depressive symptoms, body mass index (BMI calculated as kg/m2), and physical frailty level (not frail/pre-frail/frail). Number of depressive symptoms were obtained from the Hospital Anxiety and Depression scale (HADS) [27]. Physical frailty status (not frail/pre-frail/frail) was derived for each participant using the Fried Phenotype guidelines [28], for more detail see Welstead et al. [29]. For the purposes of our longitudinal analysis, this was recoded as a binary variable (not frail versus pre-frail/frail).

### Statistical analysis

Comparisons of the predictors across different MCI transition states were assessed by ANOVAs and Pearson’s Chi-squared tests. Multinomial logistic regression models were fit in order to assess the effect of various predictors on the likelihood of fitting into one of three potential outcomes between T1 and T2 compared to the reference group of those who remained cognitively healthy. Outcomes were: 1) Participant remains categorized as having MCI, 2) Participant reverts from MCI to healthy cognition, 3) Participant transitions from healthy cognition to MCI. Participants may have progressed to dementia over follow-up; however, they would then not have been eligible to take part in the next data wave, or if they did, they would be excluded from our analyses. Participants who withdrew from the study were not assessed as an outcome as the reason for withdrawal was not known. Risk of fitting in to each of these categories was calculated in a baseline model controlled for age and sex before computing a full model with adjustment for all covariates. Associations between the covariates were below 0.4, indicating that that the variance of the model’s regression coefficient was not inflated by multicollinearity. Goodness of model fit was assessed using McFadden’s R-squared [30] and found to have a value of 0.31, between the typical ‘very good fit range’ of 0.2 to 0.4. All analyses were conducted in R version 3.5.3 [31].

## RESULTS

Of 697 participants at T1, 127 were excluded because of missing data, and three were excluded as they had developed dementia. MCI was coded for 567 participants at T1 and 341 at T2, but 49 of those individuals did not have MCI status at T1 so the final number of participants with MCI status ascertained at both time points was 292. At T1, 41 (14%) participants were categorized as having MCI, while at T2 there were 56 (19%) participants with MCI. MCI status at each time point for the 292 participants is reported in Table 1.

**Table 1:** MCI status at T1 and T2 for participants who completed both time points

		T2 MCI status		
		Healthy cognition	MCI	*Total*
T1 MCI status	Healthy cognition	215	36	251
	MCI	21	20	41
	*Total*	236	56	292

**Fig. 2:**
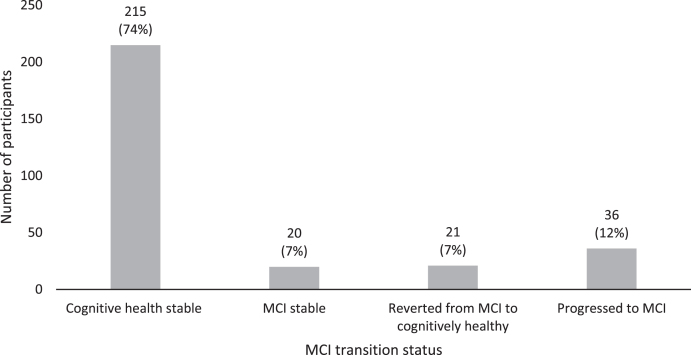
MCI transition rates over six-year follow-up (T1 and T2).

74%(*n* = 215) remained cognitively healthy, 7%(*n* = 20) remained MCI, 7%(*n* = 21) reverted from MCI to healthy cognition, and 12%(*n* = 36) transitioned to MCI. Figure 2 illustrates these transition rates.

Associations between covariates and MCI transition status were significant for number of depressive symptoms and cardiovascular history (see Table 2). *Post-hoc t*-tests tested the difference in cardiovascular history between specific MCI transition groups. Individuals who remained cognitively healthy between T1 and T2 had significantly lower rates of cardiovascular disease (33.5%of participants) compared to those who remained MCI at both time points (60.0%of participants) (*t*(233) = 2.38, *p* = 0.018). Similarly, those who remained cognitively healthy had significantly fewer T1 depressive symptoms than those who remained MCI stable (*t*(233) = 15.49, *p* < 0.001), and those who reverted from MCI to healthy cognition (*t*(233) = 2.60, *p* < 0.01).

**Table 2:** Predictor variables according to MCI Transition Status

Variables	Maintained healthy cognition status (N = 215)	Maintained MCI status (N = 20)	Transitioned from healthy cognition to MCI (N = 36)	Reverted from MCI to healthy cognition (N = 21)	*p*
Age at T1, mean (SD)	76.24 (0.68)	76.13 (0.69)	76.25 (0.72)	75.99 (0.74)	0.389^1^
Sex, *n* (%)					0.233^2^
Male	100 (46.5%)	13 (65.0%)	21 (58.3%)	12 (57.1%)
Female	115 (53.5%)	7 (35.0%)	15 (41.7%)	9 (42.9%)
Age 11 cognitive function, mean (SD)	2.50 (11.06)	3.17 (11.81)	1.39 (10.92)	2.61 (10.66)	0.943^1^
Missing data	13	2	3	2
Years of education, mean (SD)	10.94 (1.16)	11.20 (1.06)	11.06 (1.26)	11.05 (1.32)	0.774^1^
Depressive symptoms, mean (SD)	2.28 (1.86)	2.75 (2.92)	2.92 (2.58)	2.47 (1.96)	0.024^1 *^
Body mass index, mean (SD)	27.52 (4.02)	26.96 (3.40)	27.67 (3.89)	27.51 (3.93)	0.920^1^
Social class, *n* (%)					0.903^2^
Professional	57 (26.6%)	7 (35.0%)	8 (23.5%)	3 (15.0%)
Managerial	80 (37.4%)	7 (35.0%)	13 (38.2%)	13 (65.0%)
Skilled non-manual	48 (22.4%)	2 (10.0%)	10 (29.4%)	3 (15.0%)
Skilled manual	25 (11.7%)	4 (20.0%)	3 (8.8%)	1 (5.0%)
Semiskilled/Unskilled	4 (1.9%)	0 (0.0%)	0 (0.0%)	0 (0.0%)
Missing data	1	0	2	1
History of cardiovascular disease, *n* (%)					0.028^2 *^
No	143 (66.5%)	8 (40.0%)	26 (72.2%)	10 (47.6%)
Yes	72 (33.5%)	12 (60.0%)	10 (27.8%)	11 (52.4%)
History of stroke, *n* (%)					0.052^2^
No	198 (92.1%)	15 (75.0%)	30 (83.3%)	18 (85.7%)
Yes	17 (7.9%)	5 (25.0%)	6 (16.7%)	3 (14.3%)
*APOE* *ɛ4* status, *n* (%)					0.056^2^
Absent	155 (76.7%)	11 (57.9%)	19 (57.6%)	15 (71.4%)
Present	47 (23.3%)	8 (42.1%)	14 (42.4%)	6 (28.6%)
Missing data	13	1	3	0
Fried Phenotype Status, *n* (%)					0.767^2^
Not Frail	108 (50.2%)	10 (50.0%)	18 (50.0%)	8 (38.1%)
Pre-Frail/Frail	107 (49.8%)	10 (50.0%)	18 (50.0%)	13 (61.9%)

^*^p < 0.05, ^**^p < 0.01, ^***^p < 0.001; ^1^Linear Model ANOVA; ^2^Pearson’s Chi-squared test.

Significant differences were noted between completers and withdrawers. Specifically, those who withdrew from the study had a lower age 11 cognitive function (completers M[SD] = 2.42 [11.01], withdrawers M[SD] = –0.02 [12.14], F(1, 529) = 5.88, *p* = 0.016), fewer years of education (completers M[SD] = 10.98 [1.17], withdrawers M[SD] = 10.61 [1.07], F(1, 565) = 15.54, *p* < 0.001), more depressive symptoms (completers M[SD] = 2.47 [1.96], withdrawers M[SD] = 3.00 [2.38], F(1, 529) = 5.88, *p* = 0.004), were more likely to come from a lower occupational social class (*χ*^2^(5) = 22.94, *p* < 0.001), and were more likely to be frail (completers = 43.2%pre-frail/frail, withdrawers = 57.8%pre-frail/frail, *χ*^2^(1) = 18.38, *p* < 0.001). Full comparisons are reported in Supplementary Table 1.

A baseline multinomial logistic regression model was fitted using sex and age as covariates and comparing three MCI transition statuses (MCI stable, healthy cognition to MCI, MCI to healthy cognition) against a reference group (remained cognitively healthy). Odds ratios and 95%confidence intervals were calculated to determine the effect of a one-unit increase in predictor variables on the odds of being in a particular MCI transition category. In the baseline model, age showed a significant association with MCI status transition, but sex did not. A fully adjusted model with all of the predictors was then computed. It showed that, compared to staying cognitively healthy, participants who were older at T1 were less likely to maintain an MCI status (OR = 0.57 [0.51–0.64], *p* < 0.001), revert from MCI to healthy cognition (OR = 0.24 [0.21–0.26], *p* < 0.001), or progress from healthy cognition to MCI (OR = 0.91 [0.84–0.99], *p* < 0.05) over the follow-up period. Furthermore, compared with remaining cognitively healthy, every unit increase in depressive symptoms significantly increased the likelihood that a participant would revert from MCI to healthy cognition (OR = 1.37 [1.05–1.80], *p* < 0.05). A history of cardiovascular disease was also shown to increase the likelihood of a participant maintaining an MCI status across follow-up (OR = 3.13 [1.01–9.70], *p* < 0.05). No further significant associations were found, individual odds ratios are reported in Table 3.

**Table 3:** Odds ratios of MCI status compared to the ‘remained cognitively healthy’ reference group

	MCI stable (*n* = 20)		Transitioned healthy cognition to MCI (*n* = 36)		Reverted MCI to healthy cognition (*n* = 21)	
Covariates	*Odds Ratios (95%CI)*	*p*	*Odds Ratios (95%CI)*	*p*	*Odds Ratios (95%CI)*	*p*
Age	0.57 (0.51–0.64)	< 0.001^***^	0.91 (0.84–0.99)	< 0.001^***^	0.24 (0.21–0.26)	< 0.001^***^
Sex (1 = male/2 = female)	1.16 (0.30–4.44)	0.83	0.79 (0.29–2.15)	0.65	1.44 (0.40–5.23)	0.58
Age 11 cognitive function	0.99 (0.95–1.03)	0.58	0.98 (0.94–1.02)	0.28	0.99 (0.95–1.03)	0.66
Years of education	1.39 (0.78–2.45)	0.26	1.18 0.75–1.87)	0.47	1.43 (0.81–2.52)	0.21
Depressive symptoms	1.15 (0.85–1.57)	0.36	1.01 (0.79–1.28)	0.96	1.37 (1.05–1.80)	0.04^*^
Body mass index	0.95 (0.80–1.11)	0.50	1.06 (0.95–1.20)	0.29	0.99 (0.86–1.14)	0.90
Social class	1.27 (0.58–2.82)	0.55	1.05 (0.56–1.97)	0.87	1.21 (0.54–2.75)	0.64
History of cardiovascular disease	1.55 (0.44–5.48)	0.50	3.13 (1.01–9.70)	0.04^*^	0.58 (0.15–2.22)	0.42
*APOE* *ɛ4* carrier	1.91 (0.55–6.65)	0.31	2.60 (0.98–6.91)	0.06	1.36 (0.36–5.20)	0.65
Fried Phenotype (non-frail versus pre-frail/frail)	1.16 (0.33–4.14)	0.82	2.00 (0.72–5.52)	0.18	1.11 (0.29–4.22)	0.88

^*^p < 0.05, ^**^p < 0.01, ^***^p < 0.001.

## DISCUSSION

In this study, we found that, of those with MCI at T1, half remained so but half returned to healthy cognition. Of those who were cognitively healthy at T1, the majority stayed this way at T2 with a smaller proportion progressing to MCI. Age, history of cardiovascular disease, and number of depressive symptoms significantly differed between MCI transition groups. The effect of age was such that those who were older at T1 were less likely to remain MCI stable, revert to healthy cognition, or progress to MCI. Higher number of depressive symptoms increased the likelihood of reverting from MCI to healthy cognition and having a history of cardiovascular disease increased the likelihood of remaining MCI stable across follow-up. We discuss next the potential reasons behind our findings and how they compare in a wider research context.

### Comparison with other literature and interpretation

Our findings of MCI progression and stability rates aligned with previous findings. While rates of reversion seem lower than in other studies, which report anywhere between 14%and 57%[16, 17, 32], this is likely due to our inclusion of cognitively healthy participants at baseline. Findings indicated that older age at T1 was a significant factor decreasing the likelihood of maintaining, reverting from, or progressing to MCI compared to remaining in cognitively healthy. This is somewhat surprising for two reasons. Firstly, the variance in T1 age is small as the LBC1936 is a narrow age cohort. Secondly, while we would expect increased age to have negative effects on MCI transition, the positive effects found do not fit with previous findings that increased age increases MCI risk [33]. Further research is required to investigate this more thoroughly.

Additional logistic regression findings showed that higher depressive symptoms at T1 increased likelihood of reverting from MCI to healthy cognition. Our results may reflect a pseudo-dementia, whereby the symptoms of depression in older age mirror those of cognitive decline. [34]. In theory, as these depressive symptoms improve, their negative cognitive impact will be ameliorated. Sugarman et al. [35] reported that successful treatment of depression increased probability of reverting from MCI to healthy cognition. Accordingly, clinical care interventions targeted at treating depression may help to reduce risk of cognitive decline. However, another interpretation of our results is to consider is that depression not cause cognitive impairment, but may exacerbate cognitive impairment by uncovering decline caused by neurodegenerative processes that had until then be unobserved [36]. Accordingly, while treating depression may provide a short reprieve, the underlying cause may remain.

The final findings from our regression analyses indicated that cardiovascular history increases the likelihood of remaining with MCI status at follow-up. This is perhaps unsurprising considering the previous research indicating that cardiovascular health issues are associated with cognitive decline [13, 37], as well as current clinical guidelines that endorse the reduction of cardiovascular risk as a method of tackling dementia risk [38].

### Strengths and limitations

This study has several limitations. Firstly, while our sample was of reasonable size, when this was broken down into different MCI transition categories the number of participants in some groups was small and potentially limited the power and accuracy of our statistical analyses. Secondly, participants with dementia were ineligible and accordingly this study was not able to consider those who progressed to or reverted from a dementia diagnosis. The dataset also lacked information on why participants withdrew from the study leaving us unable to differentiate between those who died from those who were diagnosed with dementia or simply withdrew without reason. Thirdly, the LBC1936 has been shown to be skewed towards those with higher socio-economic status [20], which could affect the results and make them less generalizable to the general population. Fourthly, cardiovascular disease was assessed categorically as a Yes/No self-report question. While self-report measures can be highly efficient, they may also introduce a level of inaccuracy when compared to using medical records or physical examination. Finally, due to a lack of cases we were unable to assess the effect of having a history of stroke on MCI transition, which is an important consideration due to previously established associations between stroke and cognitive impairment [39]. This study also had strengths and benefitted from considering both progressions to, and reversions from MCI. Most previous research focusses on one of these two processes rather than both simultaneously and subsequently may be missing salient information. Additionally, this study utilized a relatively long follow-up period of six years, allowing for a better understanding of the long-term fluidity of MCI, and considered factors such as childhood cognitive function that have not previously been investigated.

### Future directions

Future research is required to delineate the predictive factors that can influence MCI transition between healthy cognition and cognitive impairment. A particularly interesting question is whether MCI progression and reversion have the same predictive factors. Our findings indicate that this may not be the case, which has important ramifications for the differential treatment of those at risk of developing MCI and those with MCI. Future research may also benefit from using additional time points to further examine the fluid nature of MCI status; it would be particularly interesting to see whether those reverting from MCI to healthy cognition remain that way over time.

## CONCLUSION

While a considerable amount of longitudinal research has investigated the factors associated with dementia risk, fewer studies have focused on the progression and reversion transitions between a healthy cognition and MCI. Our findings indicate a slow but consistent increase in MCI rates over a six-year period in the LBC1936. Despite the overall increase, we also find that around 4%of participants actually show an improvement over time and revert from MCI to healthy cognition. Higher instances of baseline depressive symptoms were associated with an increased likelihood to revert from MCI to healthy cognition over follow-up. These findings potentially indicate the important role of early identification and treatment of depression in clinical care to help address cognitive decline. Our findings add to previous literature and highlight the potential for a two-pronged approach to addressing MCI: 1) effectively designing prevention strategies that target risk factors between healthy cognition and progression to MCI, and 2) implementing interventions for those living with MCI to facilitate reversion to healthy cognition. Future research should continue exploring these factors and the fluid nature of MCI in older adults.

## Supplementary Material

Supplementary Material
