# Supplementary material for: Predictors of Mild Cognitive Impairment Stability, Progression, or Reversion in the Lothian Birth Cohort 1936
Source: J Alzheimers Dis. 2021 Mar 9;80(1):225–32. doi: 10.3233/JAD-201282 (PMC8075399; doi:10.3233/JAD-201282)
Supplement: Supplementary Material [file jad-80-jad201282-s001.pdf]

Supplementary Material

Predictors of Mild Cognitive Impairment Stability, Progression, or Reversion in the Lothian Birth Cohort 1936

Each of the MCI criteria were established by following the National Institute on Aging-Alzheimer's Association (NIA-AA) workgroups on diagnostic guidelines for Alzheimer's disease [1]:

1. *Concern regarding a change in cognition*: In order to retrospectively assess this criterion, we identified a question in the LBC1936 that has also been used in previous MCI classifications [2]. The self-reported question asks 'Do you have any problems with your memory?'

2. *Impairment in one of more cognitive domains*: Due to the longitudinal nature of the LBC1936, it is possible to assess cognitive change over time. Consistent with the NIA-AA workgroup guidelines [1], we defined the cognitive impairment criterion as: At least one cognitive domain score scoring ≥1.5 standard deviations lower than the mean **OR** A decline below the 10th percentile on one test since the previous wave **AND** below the 20th percentile on two tests since the previous wave **AND** a decline below the 20th percentile on one test since wave 1. These percentile cut offs were chosen to reflect evidence of a lower than expected performance in cognitive ability since their previous visit given their age. The cut offs are consistent with previous research [3]. We purposefully included both a cross-sectional measure of cognitive impairment as well as a measure of cognitive decline that captures those with slow decline (since wave 1) as well as a more rapid decline (since previous wave). The following cognitive tests were used: Wechsler Adult Intelligence Scale III (WAIS-III) - Symbol Search, Digit Symbol Coding, Matrix Reasoning, Letter-Number Sequencing, Block Design, and Wechsler Memory Scale III (WMS-III) Logical Memory I & II [4].

3. *Preservation of independence in functional abilities*: In the LBC1936 we have the Townsend Disability Scale [5] which scores participants out of 18, with higher scores reflecting

greater disability. The criterion is met if the participant has little decline (≤1 point negative change) since the previous wave on the Townsend Disability Scale, reflecting a general maintenance in their independence of function. Alternatively, the participant can also meet the criteria if they answered ‘No’ to the question ‘Are your memory problems affecting your life’.

4. *No diagnosis of dementia*: The fourth and final criterion is met if the participant does not self-report a diagnosis of dementia (or for the sample who have been assessed clinically, do not have a formal diagnosis of dementia made by a research doctor) **AND** scores ≥24 on the MMSE.

REFERENCES

[1] Albert MS, DeKosky ST, Dickson D, Dubois B, Feldman HH, Fox NC, Gamst A, Holtzman DM, Jagust WJ, Petersen RC (2011) The diagnosis of mild cognitive impairment due to Alzheimer's disease: recommendations from the National Institute on Aging-Alzheimer's Association workgroups on diagnostic guidelines for Alzheimer's disease. *Alzheimers Dement* **7**, 270-279.

[2] Matthews FE, Stephan BC, Bond J, McKeith I (2007) Operationalisation of mild cognitive impairment: a graphical approach. *PLoS Med* **4**, 1615-1619.

[3] Knopman DS, Gottesman RF, Sharrett AR, Wruck LM, Windham BG, Coker L, Schneider AL, Hengrui S, Alonso A, Coresh J (2016) Mild cognitive impairment and dementia prevalence: the atherosclerosis risk in communities neurocognitive study. *Alzheimers Dement* **2**, 1-11.

[4] Psychological Corporation (1997) *WAIS-III/WMS-III Technical Manual*.

[5] Townsend P (1979) *Poverty in the United Kingdom: A Survey of Household Resources and Standards of Living*, University of California Press.

Supplementary Table 1. Comparisons between completers and withdrawers.

Variables	Withdrew from study (n=275)	Completed both time points (n=292)	p
Age at T1, mean (SD)	76.278 (0.652)	76.217 (0.690)	0.280[1]
Sex, n (%)			0.109[2]
Male	156 (56.7%)	146 (50.0%)	
Female	119 (43.3%)	146 (50.0%)	
Age 11 cognitive function, mean (SD)	-0.021 (12.137)	2.415 (11.011)	0.016*[1]
Missing data	16	20	
Years of education, mean (SD)	10.611 (1.066)	10.983 (1.174)	< 0.001***[1]
Depressive symptoms, mean (SD)	3.000 (2.380)	2.473 (1.959)	0.004**[1]
Missing data	1	0	
Body mass index, mean (SD)	27.913 (4.811)	27.512 (3.930)	0.277[1]
Missing data	2	0	
Social class, n (%)			< 0.001***[2]
Professional	42 (15.4%)	75 (26.0%)	
Managerial	111 (40.7%)	113 (39.2%)	
Skilled non-manual	50 (18.3%)	63 (21.9%)	
Skilled manual	55 (20.1%)	33 (11.5%)	
Semiskilled/Unskilled	15 (5.5%)	4 (1.4%)	
Missing data	2	4	
History of cardiovascular disease, n (%)			0.203[2]
No	190 (69.1%)	187 (64.0%)	
Yes	85 (30.9%)	105 (36.0%)	
History of stroke, n (%)			0.514[2]
No	241 (87.6%)	261 (89.4%)	
Yes	34 (12.4%)	31 (10.6%)	
APOE ε4 status, n (%)			0.324[2]
Absent	179 (68.8%)	200 (72.7%)	
Present	81 (31.2%)	75 (27.3%)	
Missing data	15	17	
Fried Phenotype Status, n (%)			< 0.001***[2]
Not Frail	86 (31.3%)	144 (49.3%)	
Pre-Frail/Frail	189 (68.7%)	148 (50.7%)	

*p<0.05, **p<0.01, ***p<0.001

[1] Linear Model ANOVA; [2] Pearson's Chi-squared test
